# Fukutin-Related Protein Resides in the Golgi Cisternae of Skeletal Muscle Fibres and Forms Disulfide-Linked Homodimers via an N-Terminal Interaction

**DOI:** 10.1371/journal.pone.0022968

**Published:** 2011-08-23

**Authors:** Maisoon Alhamidi, Elisabeth Kjeldsen Buvang, Toril Fagerheim, Vigdis Brox, Sigurd Lindal, Marijke Van Ghelue, Øivind Nilssen

**Affiliations:** 1 Division of Child and Adolescent Health, Department of Medical Genetics, University Hospital of North-Norway, Tromsø, Norway; 2 Department of Clinical Medicine – Medical Genetics, University of Tromsø, Tromsø, Norway; 3 Department of Pathology, University Hospital of North-Norway, Tromsø, Norway; 4 Institute of Medical Biology, University of Tromsø, Tromsø, Norway; Consejo Superior de Investigaciones Cientificas, Spain

## Abstract

Limb-Girdle Muscular Dystrophy type 2I (LGMD2I) is an inheritable autosomal, recessive disorder caused by mutations in the FuKutin-Related Protein (FKRP) gene (*FKRP*) located on chromosome 19 (19q13.3). Mutations in *FKRP* are also associated with Congenital Muscular Dystrophy (MDC1C), Walker-Warburg Syndrome (WWS) and Muscle Eye Brain disease (MEB). These four disorders share in common an incomplete/aberrant O-glycosylation of the membrane/extracellular matrix (ECM) protein α-dystroglycan. However, further knowledge on the FKRP structure and biological function is lacking, and its intracellular location is controversial. Based on immunogold electron microscopy of human skeletal muscle sections we demonstrate that FKRP co-localises with the middle-to-trans-Golgi marker MG160, between the myofibrils in human *rectus femoris* muscle fibres. Chemical cross-linking experiments followed by pairwise yeast 2-hybrid experiments, and co-immune precipitation, demonstrate that FKRP can exist as homodimers as well as in large multimeric protein complexes when expressed in cell culture. The FKRP homodimer is kept together by a disulfide bridge provided by the most N-terminal cysteine, Cys6. FKRP contains N-glycan of high mannose and/or hybrid type; however, FKRP N-glycosylation is not required for FKRP homodimer or multimer formation. We propose a model for FKRP which is consistent with that of a Golgi resident type II transmembrane protein.

## Introduction

Defects of α-dystroglycan (α-DG) O-glycosylation are associated with several forms of inheritable muscular dystrophies (Limb Girdle Muscular Dystrophy type 2I; LGMD2I), of which some are congenital (Congenital Muscular Dystrophy type 1C; MDC1C), and some are associated with brain (Fukyama Congenital Muscular Dystrophy; FCMD, Walker-Warburg Syndrome; WWS) and eye abnormalities (Muscle Eye Brain disease; MEB) [1]. α-DG is a component of the dystrophin-glycoprotein complex (DGC) and contains multiple sites for O-linked glycosylation [2], [3]. Proper O-glycosylation of α-DG is crucial for its interaction with the extracellular laminin-α2 and agrin in muscle, and neurexin in brain [4], [5], [6]. α-DG hypoglycosylation precludes these interactions and the disruption of the link between these extracellular components and the actin cytoskeleton, is thought to be part of the molecular pathogenesis of the muscular dystrophy phenotype in LGMD2I and the additional brain involvement seen in WWS and MEB [7]. These disorders are collectively known as dystroglycanopathies, and they can be caused by mutations in any one of six different genes encoding known or putative glycosyltransferases or phosphotransferases. These genes are *POMGnT1*, encoding protein *O*-linked mannose *ß*-1,2-*N* acetylglucosaminyl-transferase 1 [8], [9], *POMT1* and *POMT2* encoding a complex that confers protein O-mannosyltransferase activity [10], [11], [12], *LARGE* [13] likely to be involved in post-phosphoryl modification of phosphorylated O-linked mannose [14], *FKTN* [15] encoding fukutin, a putative phosphoryl ligand transferase [16] and, finally, the fukutin related protein gene, *FKRP* [17], [18], encoding a 495 aa polypeptide (human FKRP; hFKRP) of unknown function.

Mutations in *FKRP* were originally reported to cause MDC1C, a severe form of congenital muscular dystrophy [17], however, later it has become clear that mutations in the *FKRP* gene might cause a wider range of phenotypes such as those of LGMD2I [18], MEB [19] and WWS [20].

FKRP has been postulated to be involved in the O-glycosylation of α-DG. This was based on the shift in α-DG molecular weight and change in band intensity seen on Western blots of muscle extracts from patients with MDC1C, using glycan dependent anti α-DG antibodies [17]. Correspondingly, immune histochemistry showed depletion of glycosylated α-DG in muscle sections from LGMD2I patients when glycan specific α-DG antibodies were used [18], but not when GT20DAG antibodies directed towards the core protein were used [21]. FKRP and its homologue Fukutin contain DXD motifs shared by some glycosyltransferases [22]. However importantly, this family of proteins also share sequence similarity with phosphoryl ligand transferases [16], [23].

Attempts to solve the intracellular localisation of FKRP have produced contradicting results. Previous studies based on immune-cytochemistry/-histochemistry on various types of cultured cells or tissue sections, from human and rodent muscle, have indicated localisation of FKRP to the endoplasmic reticulum (ER) [24], [25], the Golgi apparatus [26], [27], [28], [29], [30] and the muscle cell sarcolemma [31].

In this work, by employing high resolution immunogold electron microscopy, we demonstrate that FKRP co-localises with the middle-to-trans Golgi marker MG160, between the myofibrils, in human *rectus femoris* muscle fibres. Furthermore, we demonstrate that FKRP can interact with itself in living cells and that FKRP can exist as a homodimer and in multimeric protein complexes. FKRP homodimer formation depends on an N-terminal interaction interface at which the dimers are covalently linked by a disulfide bridge provided by Cys6, preceding a putative N-terminal trans-membrane sequence motif. FKRP contains two putative N-glycosylation sites. Both are occupied with high mannose/hybrid type of oligosaccharides. However, N-glycosylation is not required for FKRP homodimer or multimer formation.

## Results

### FKRP co-localises with the Golgi marker MG160, between the myofibrils of human skeletal muscle fibres

**Figure 1:**
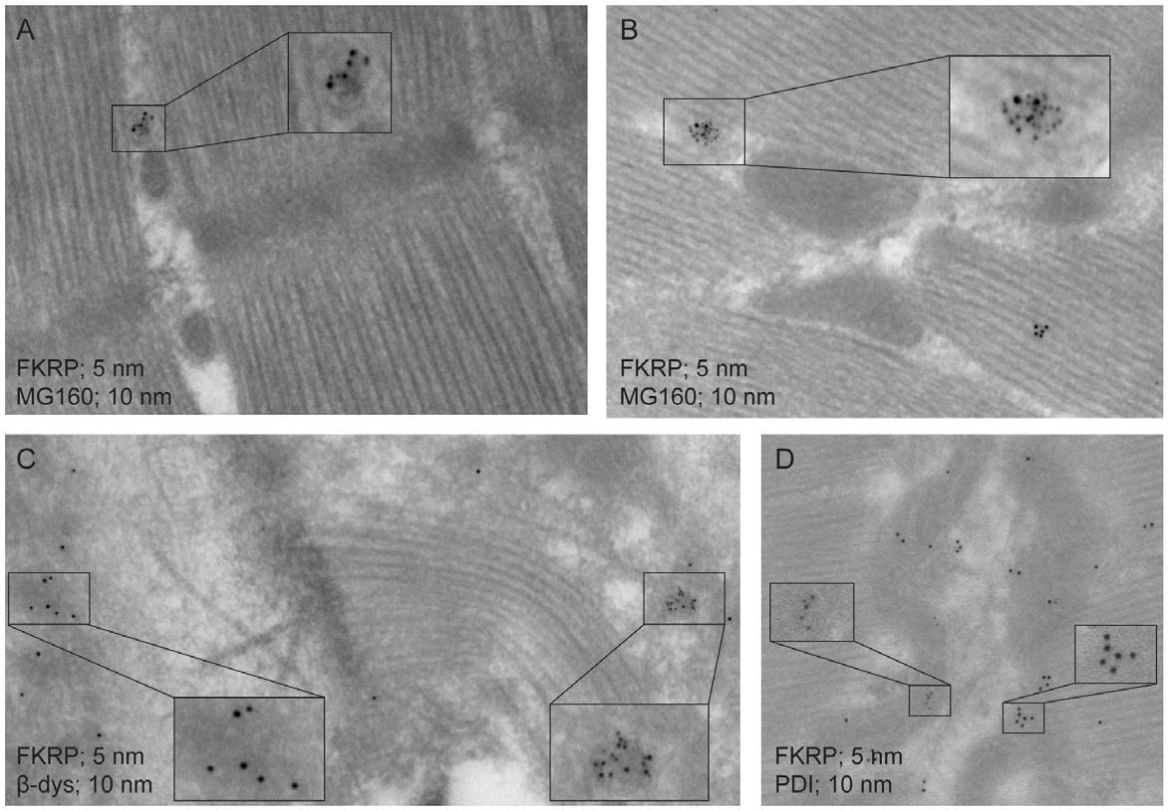
FKRP subcellular localisation in skeletal muscle, determined by double EM immunogold labelling. FKRP was detected with a mixture of FKRP207 and FKRP208 primary antibodies followed by labelling with goat anti-rabbit antibody, conjugated with 5 nm gold particles (A, B, C and D). Subcellular marker antibodies were, anti MG160 (Golgi) (A, B), anti ß-dys (sarcolemma) (C) and anti PDI (endoplasmic reticulum) (D). Secondary antibody for detection of subcellular marker proteins was goat anti-mouse antibody, conjugated with 10 nm gold particles. Selected areas (framed) were enlarged by 200%.

To investigate the *in vivo* localisation of endogenous FKRP in muscle cells, separate double immunogold labelling experiments were performed on human *rectus femoris* longitudinal sections, using antibodies against FKRP and protein markers specifying either the sarcolemma (anti ß-dystroglycan), Golgi (anti MG160) or endoplasmic reticulum (ER) (anti PDI). Overall, the 5 nm gold particles, specific to FKRP, clustered (2–15 grains) at discrete locations in the myofibrillar core, mainly between the myofibrils (Fig. 1 A, B). Five-nm particles were associated with vesicle- or cisternae-like structures and often they were found in close proximity with mitochondria. Their locations were distinctly different from those of ß-dystroglycan specific 10 nm gold particles which were found to be scattered continuously along the muscle cell membrane (Fig. 1C). Similar to signals specific to FKRP, PDI specific signals were detected mainly between myofibrils but they were also frequently associated with terminal sarcoplasmic reticulum (SR) cisternae, neighbouring T-tubule like structures (Fig. 1 D). However, FKRP and PDI specific signals were usually not associated, but rather they appeared as spatially separated clusters of 5 nm and 10 nm particles, respectively. In contrast, FKRP specific 5 nm particles co-localised and, in fact, overlapped with clusters of 10 nm particles specific to the Golgi marker MG160 (Fig. 1A and B). The MG160 marker was previously found to be located in the middle to trans-cisternae of the Golgi complex in *soleus* muscle cells of adult rats [32]. No co-localisation of 5 nm and 10 nm particles was observed when omitting one of the primary antibodies, in a series of control experiments as explained in the Materials and Methods. Hence, we conclude that the Golgi middle-to-*trans* cisterna is the location of FKRP in human adult *rectus femoris* muscle cells.

### FKRP is part of multimeric complexes that are sensitive to reduction by DTT

**Figure 2:**
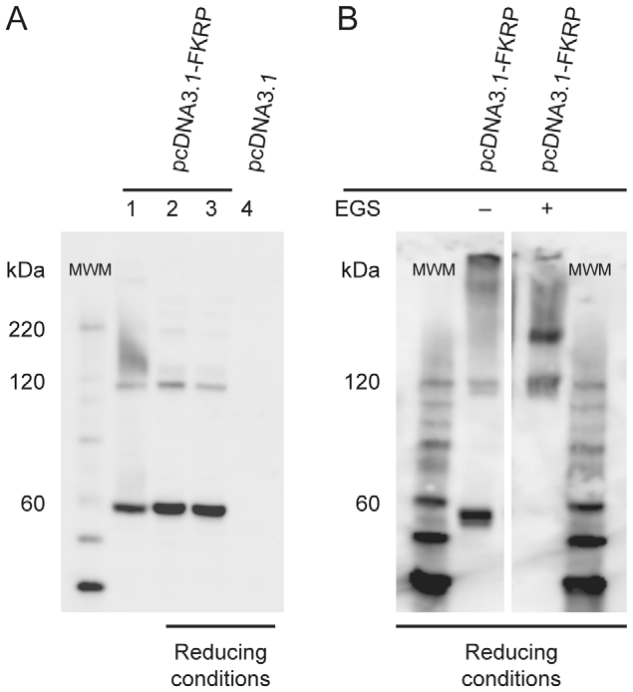
Recombinant FKRP forms multimers in mammalian cells. COS-7 and BHK-21 cells were transfected with pcDNA3.1-FKRP or empty vector (pcDNA3.1). Forty-eight hrs after transfection cells were solubilised in lysis buffer with protease inhibitor. A) The cleared COS-7 lysate was either kept untreated (lane 1) or reduced with 400 mM DTT at RT for 30 min (lane 2) or Sample Reducing agent at 99°C for 5 min (lanes 3 and 4) and subsequently subjected to (4–12%) SDS-PAGE and Western blot analysis. Lanes 1–3: lysates from pcDNA3.1-FKRP transfections. Lane 4: lysate from pcDNA3.1 transfection serving as negative control. B) A cleared BHK-21 lysate was treated with 10 mM EGS at RT for 30 min and quenched with 35 mM Tris, pH 7.5 for 15 min. The EGS treated sample and the accompanying untreated control sample were reduced with Sample Reducing agent at 99°C for 5 min and subjected to SDS-PAGE and Western blot analysis. In the above experiments FKRP was detected using FKRP207 antibodies. MWM is molecular weight marker.

Western blot analysis of lysates from FKRP expressing cells, performed under reducing conditions, revealed a prominent FKRP specific band migrating as the size of a monomer (∼58 kDa), as well as an additional band (∼116 kDa) of lower intensity, approximately twice the size as that expected for the FKRP monomer (Fig. 2A, lanes 2 and 3). Discrete bands of higher molecular weight were also observed. Under non-reducing conditions an additional smeared higher molecular weight band ranging from ∼120 to ∼220 kDa as well as a distinct band of >∼450 kDa were detected (Fig. 2A, lane 1). The involvement of FKRP in these high molecular weight structures was further investigated by adding the cross-linker Ethylene glycol-bis(SuccinimidylSuccinate) (EGS) immediately after cell lysis. EGS is a homobifunctional, DTT insensitive, protein cross-linker with a spacer arm of 16.1 Å that can covalently link interacting proteins. As shown in Figure 2B, upon cross-linking the FKRP monomer disappeared and only higher molecular weight bands could be detected. Distinct protein bands appeared at ∼116 kDa and ∼170–180 kDa and a smear of bands ranging in molecular weights from 180 kDa to several hundred kDa were observed.

We conclude that FKRP is part of larger multimeric protein complexes that range in size from the molecular weight of a dimer to several hundred kDa. Some of these complexes are sensitive to reduction by DTT and, accordingly, their components are likely linked by disulfide bridges (Fig. 2A and B).

### FKRP interacts with itself in living cells

The presence of a ∼116 kDa band *per se* is suggestive but not evidence of an FKRP dimer. The ∼116 kDa band as well as higher molecular weight protein bands might reflect covalent interactions of FKRP with other proteins. Therefore, we investigated if FKRP had the capacity to interact with itself in a pairwise yeast two-hybrid experiment followed by a co-immune precipitation (Co-IP) experiment.

**Figure 3:**
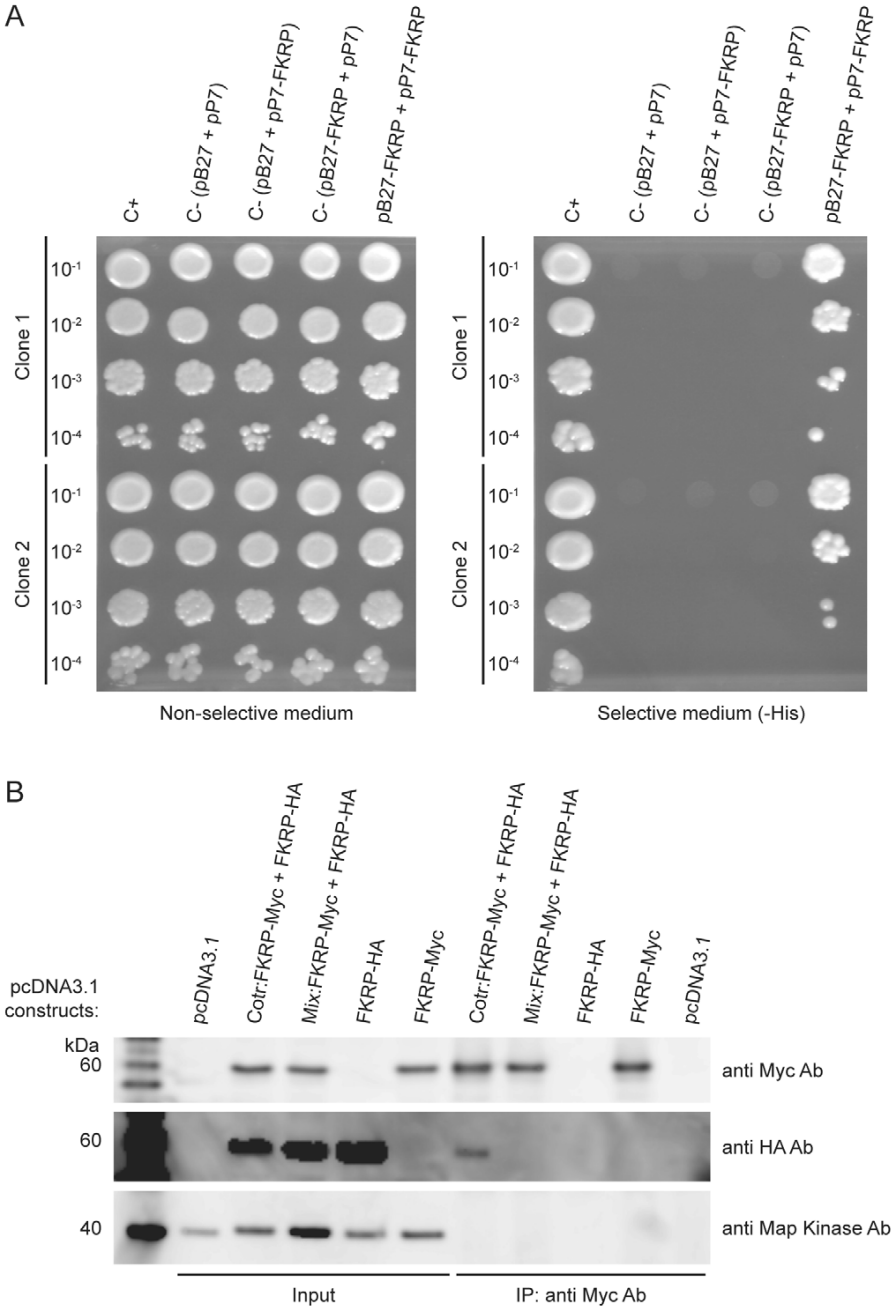
FKRP self-interaction as demonstrated by pair wise Y2H analysis and CO-IP experiments. A) Diploid yeast cells containing both bait construct, pB27-FKRP (N-LexA-FKRP32-494-C), and prey construct, pP7-FKRP (N-GAL4-FKRP32-494-C), were obtained by mating and spotted, at the dilutions indicated, onto non-selective media lacking Trp and Leu (left panel) and selective media lacking Trp, Leu and His (right panel). Negative controls contained empty bait and pray vectors, pB27 and pP7, or pB27 and pP7 in combination with prey and bait constructs, respectively. Positive control (C+) contained human SMAD3 as bait (GI:5174512) and Human SMURF1 as prey (GI:31317291) as explained in Materials and Methods. B) Anti-Myc antibody was employed to precipitate FKRP-Myc fusion proteins from COS-7 cell lysates. The lysates were prepared by pcDNA3.1-FKRP-Myc/pcDNA3.1-FKRP-HA co-transfection (Cotr) or solo transfections of pcDNA3.1-FKRP-Myc and pcDNA3.1-FKRP-HA. An additional sample was prepared by mixing pcDNA3.1-FKRP-Myc and pcDNA3.1-FKRP-HA lysates followed by incubation at RT for 30 min (Mix). All cell lysates used for Co-IP were prepared in the presence of 5 mM NEM. Lysates from pcDNA3.1 and pcDNA3.1-FKRP-HA solo transfections served as negative controls whereas lysates from pcDNA3.1-Myc transfected cells served as positive control for anti-Myc based immune precipitation. Input samples and precipitates were subjected to (4–12%) SDS-PAGE, under reducing conditions, followed by Western blot analysis. On separate blots FKRP-Myc and FKRP-HA were detected with anti-Myc and anti-HA antibodies, respectively. To asses the stringency of the of the Co-IP experiment, one of the blots (anti-HA) was stripped and assayed for endogenous MAPK with anti-MAPK antibody (lower panel).

The coding sequence for amino acids 32-494 of human FKRP was cloned into the bait vector pB27 in-frame with the coding sequence of LexA DNA binding domain (DBD): (pP27 (N-LexA-FKRP32-494-C)) and into the pray vector, p7, in frame with the Gal4 activation domain (AD) (pP7 (N-GAL4 -FKRP32-494-C)). Diploid yeast cells containing both bait and prey expression constructs were spotted onto non-selective and selective media along with appropriate controls, as explained in the Materials and Methods. As displayed in the left panel of Figure 3A, the control experiment demonstrated that all clones tested contained both the prey and bait vector, a requirement for growth on media lacking Leu and Trp (DO-2). However, only FKRP32-494 in bait-prey combination allowed growth on triple minus medium (DO-3) selecting for expression of the *HIS3* reporter gene (Fig. 3A, right panel). This result demonstrates that FKRP32-494 self-interaction can take place in yeast cells.

To examine if the FKRP-FKRP interaction, detected by cell growth in yeast two-hybrid assays, also occurs in mammalian cells we co-expressed full length FKRP, C-terminally tagged with HA (FKRP-HA) and Myc (FKRP-Myc) epitopes, in COS-7 cells. The cell lysates were prepared in the presence of 5 mM *N*-ethylmaleimide (NEM); an SH-group alkylating agent which modifies free SH groups of cysteines and prevents aberrant post-lysis SH-mediated protein association.

As seen in Figure 3B, FKRP-HA was co-precipitated with anti Myc antibodies, but only from a lysate originating from cells co-transfected with both FKRP-HA and FKRP-Myc expressing plasmids. FKRP-HA was not co-precipitated with FKRP-Myc from a mixture of lysates originating from separate solo transfections of FKRP-HA and FKRP-Myc carrying expression plasmids. This result demonstrates that FKRP self interaction can take place *in vivo*, in mammalian cells.

### The two FKRP N-glycosylation sites are occupied with high mannose and/or hybrid oligosaccharides

**Figure 4:**
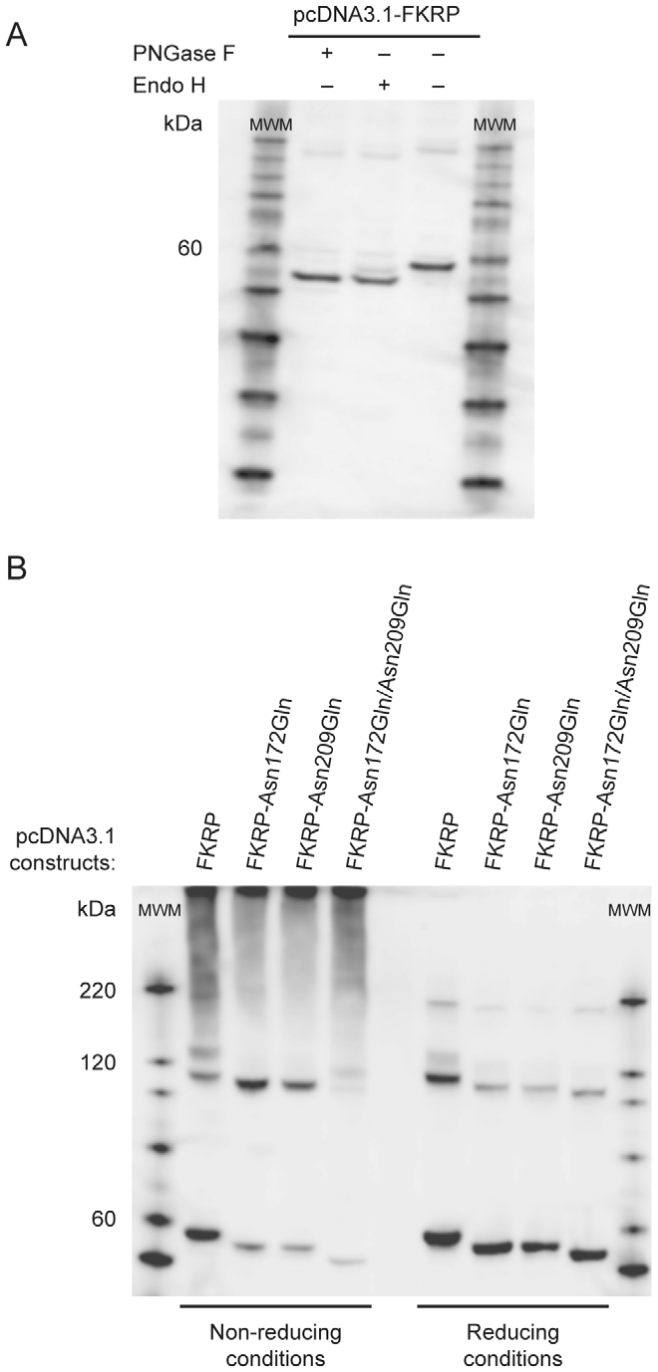
FKRP dimer and multimer formation is not dependent on N-glycosylation. Cells were transfected with pcDNA 3.1-FKRP. Forty-eight hrs after transfection cells were solubilised in lysis buffer with protease inhibitor. A) A cleared BHK-21 lysate was treated with either PNGase F or Endo H as explained in Materials and Methods and subjected to (4–12%) SDS-PAGE under reducing conditions, followed by Western blot analysis. An untreated sample served as control. B) COS-7 cells were transfected with mutant pcDNA3.1-FKRP constructs, in which either one or both asparagines (Asn) involved in N-glycosylation had been replaced with glutamine (Gln). The lysates were prepared in the presence of 5 mM NEM and subjected to either non-reducing (left panel) or reducing SDS-PAGE (330 mM DTT, RT for 30 min) (right panel), followed by Western blot analysis. Antibody FKRP207 was used for the detection of FKRP in these experiments.

Based on its primary amino acid sequence, human FKRP has a predicted molecular weight of 54.6 kDa (http://www.scripps.edu/~cdputnam/protcalc.html). However, initial Western blot analysis demonstrated a molecular weight of ∼58 kDa both under non-reducing and reducing conditions (Fig. 2A). This suggested that FKRP contains a post-translational modification of approximately 3.5 kDa. Human FKRP (as well as FKRP from any other species known) contains two putative N-glycosylation sites; AsnValSer (NVS) and AsnLeuSer (NLS) at amino acid positions 172 and 209 (hFKRP), respectively. In order to assess the composition of the FKRP modification, protein lysates obtained from BHK-21 cells transfected with human FKRP expressing plasmid, were treated separately with the N-glycan specific glycosidases, PNGase F and Endo H. Both PNGase F and Endo H treatment created a shift in the molecular weight of FKRP, corresponding to ∼3.5 kDa (Fig. 4A), equivalent to the expected molecular weight of two N-linked glycans. A similar experiment using lysates from transfected COS-7 cells gave identical results (not shown). This demonstrates that upon ectopic FKRP expression in BHK-21 and COS-7 cells, both N-glycosylation sites are occupied with N-linked glycans. Furthermore, since these glycans are sensitive to Endo H digestion they must be composed of high mannose and/or hybrid type oligosaccharides.

### FKRP self interaction does not depend on N-glycosylation

To examine if FKRP self interaction is dependent on N-glycosylation the two FKRP N-glycosylation sites were altered by replacing asparagines (N, Asn) at amino acid positions 172 and 209 with glutamines (Q, Gln). By *in vitro* mutagenesis three mutant pcDNA3.1-FKRP expressing constructs were made; FKRP-Asn172Gln, FKRP-Asn209Gln and double mutant FKRP-Asn172Gln/Asn209Gln. Mutant constructs were transfected into COS-7 cells and the resulting lysates were prepared in the presence of 5 mM NEM. The lysates were subjected to Western blot analysis both under non-reducing as well as under reducing conditions. As shown in Figure 4B, removal of both N-glycosylation sites created shifts in the FKRP molecular weight corresponding to what was seen in de-glycosylation experiments, using PNGase F and Endo H (Fig. 4A). Hence, these results confirm that recombinant FKRP is indeed occupied by two N-glycans. Furthermore, the corresponding molecular weight reduction of ∼7 kDa for the ∼116 kDa band is consistent with the above conclusion that FKRP is forming a homodimer. Removal of the FKRP N-glycosylation sites did not affect the capacity of FKRP to form dimers and multimers (Fig. 4B). Thus, we conclude that FKRP dimer and multimer formation is independent of N-glycosylation.

### FKRP-FKRP dimer formation depends on an N-terminal interaction interface and a Cys6-Cys6 disulfide linkage

**Figure 5:**
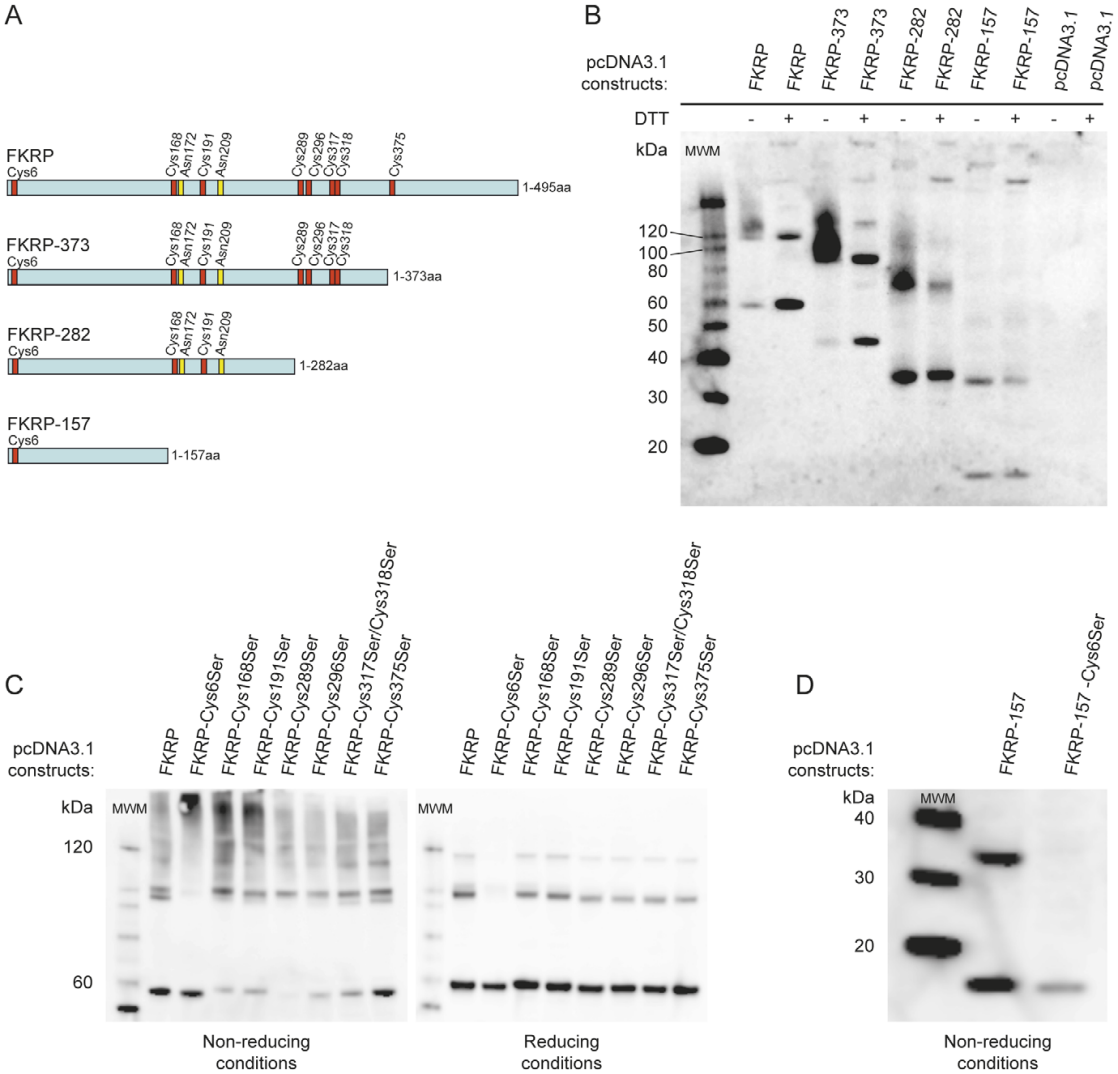
FKRP homodimer formation depends on a Cys6-Cys6 disulfide bridge. A) Schematic presentation FKRP C-terminal deletion constructs. FKRP-373, FKRP-282 and FKRP-157 denote the length (aa) of the mutant construct. Red vertical bars show Cys (C) positions whereas yellow vertical bars indicate the positions of putative N-glycosylation sites Asn-X-Thr/Ser (N-X-T/S). In the following experiments COS-7 cells were transfected with various pcDNA3.1 constructs and cleared lysates were prepared 48 hrs post transfection in the presence of 5 mM NEM. In all these experiments FKRP was detected with primary antibody FKRP207. B) Cleared lysates from FKRP C-terminal deletion mutants were either left untreated or subjected to reduction (400 mM DTT, at RT for 30 min), followed by (4–12%) SDS-PAGE and Western blot analysis. C) Cleared lysates from FKRP Cys→Ser substitution mutants were either left untreated (left panel) or subjected to reduction (400 mM DTT, at RT, for 30 min) (right panel), followed by (4–12%) SDS-PAGE and Western blot analysis. Differences in migration between monomeric forms as well as between dimeric forms (5C) likely represent different FKRP conformations resolved by SDS-PAGE. Such conformational differences might be induced by Cys→Ser mutations as some of the mutant Cys residues must be expected to be involved in intra-molecular disulfide bridges. D) COS-7 lysates from deletion construct FKRP-157, and the Cys6Ser mutant thereof, were subjected to SDS-PAGE under non-reducing conditions, followed by Western blot analysis.

In order to identify the FKRP-FKRP interaction interface we constructed C-terminal truncation mutants of FKRP: FKRP-373, FKRP-282 and FKRP-157 (Fig. 5A) of which the numbers denote the positions of the last amino acids in the truncated FKRP polypeptides. pcDNA3.1-FKRP truncation clones were expressed in COS-7 cells and the resulting lysates were prepared in the presence of 5 mM NEM and subsequently subjected to Western blot analysis. As shown in Figure 5B, FKRP truncation clones were expressed at levels comparable to that of full length FKRP, except FKRP-157 which repeatedly showed somewhat reduced expression levels. All FKRP truncation mutants, even FKRP-157, retained the property of forming dimers. However, in contrast to FKRP-157, full length FKRP, FKRP-282 and FKRP-373 also produced additional bands seen as smears under non-reducing conditions and distinct high MW bands under reducing conditions. These bands represent multimeric, FKRP containing, protein complexes (Fig. 5B). Together, these results indicate that the interaction interface responsible for homodimer formation, is located in the N-terminal one third of FKRP and, furthermore, that FKRP multimer formation depends on additional protein segments that extend beyond residue 157 and into the C-terminal two third of the FKRP polypeptide.

The FKRP polypeptide contains eight cysteines. These are located at amino acid residues 6, 168, 191, 289, 296, 317, 318 and 375. To examine the role of these cysteines in the formation of FKRP dimers and multimers, cysteine (Cys, C) to serine (Ser, S) substitutions were introduced to FKRP by *in vitro* mutagenesis (Fig. 5A). Transfected COS-7 cells were lysed with lysis buffer containing 5 mM NEM and subjected to Western blot analysis under both protein non-reducing and reducing conditions. Cys to Ser substitutions did not affect FKRP expression levels in COS-7 cells (Fig. 5C).

As displayed in Figure 5C, under both non-reducing and reducing conditions, the mutant FKRP-Cys6Ser behaved distinctly different from the *wild type* FKRP and the other FKRP Cys→Ser mutants. Under non-reducing conditions FKRP-Cys6Ser expressed only the ∼58 kDa monomer as well as a strong band of very high molecular weight (>500 kDa). In contrast, the *wild type* FKRP and the other FKRPCys-Ser mutants expressed both monomers (albeit FKRP-Cys289Ser only weakly) and dimers of ∼116 kDa, as well as smeared bands ranging in molecular weights from ∼180 kDa to several hundred kDas (Fig. 5C, left panel). Furthermore, as the only mutant, FKRP-Cys6Ser expressed exclusively FKRP monomers under reducing conditions. For *wild type* FKRP, as well as for other FKRP Cys→Ser mutants, the relative amount of monomer increased at the expense of higher order multimers. However, in contrast to FKRP-Cys6Ser, distinct bands corresponding to ∼116 kDa and ∼180 kDa were clearly visible (Fig. 5C, right panel).

In summary; as the only mutant, FKRP-Cys6Ser failed to express the FKRP homodimer band of ∼116 kDa under non-reducing, as well as under reducing conditions. Hence, we conclude that the FKRP homodimer is kept together by an intermolecular disulfide bridge provided by Cys6 (Cys6-Cys6). The role of Cys6 in dimer formation was confirmed by the observation that FKRP-157-Cys6Ser, but not FKRP-157, failed to form dimers, even under non-reducing conditions (Fig. 5D).

The involvement of other cysteines in FKRP dimer formation was not evident; however, their participation in high molecular weight structures is very likely since higher order multimers, above 180 kDa, were clearly sensitive to reduction by DTT (Fig. 5C). Their composition and the role of FKRP in these larger protein complexes remain to be investigated.

## Discussion

### FKRP subcellular localisation in muscle cells

Previous attempts to determine the intracellular localisation of both endogenous and recombinant FKRP have been carried out using immune cytochemistry on transfected cells and immune histochemistry on tissue sections from heart or skeletal muscle [24], [25], [26], [27], [28], [29], [30], [31]. General problems in protein localisation studies are mislocalisation, because of protein over-production in transfected cells, and/or lack of sensitivity or sufficient microscopic resolution when studying complex cell types such as those of skeletal muscle. This prompted us to assess endogenous muscle FKRP localisation at maximum resolution using immunoelectron microscopy on ultrathin sections of human *rectus femoris* muscle. A mixture of two polyclonal anti-FKRP antibodies, corresponding both to the N-terminal region and the C-terminal region of the FKRP polypeptide, showed co-localisation and overlap with the Golgi marker MG160, between myofibrils, in human skeletal muscle fibres. Perinuclear localisation, or co-localisation with ER-lumen marker, PDI, or sarcolemma marker, β-dystroglycan, was excluded. Hence, in accordance with the location of MG160 [32] these results provide ultra structural evidence for FKRP localisation in the middle-to-trans-cisternae of the Golgi complex.

Some of the previous attempts to identify the intracellular localisation of FKRP have been based on immune cytochemistry on transfected cell lines of various origins. In this regard the Golgi localisation of endogenous FKRP reported here is in accordance with the findings of Esapa *et al* (2002 and 2005) [26], [27], Keramaris-Vrantsis *et al* (2007) [29] and Lu *et al* (2010) [30], however, they conflict the findings of Matsumoto *et al* (2004) [24] who found ectopically expressed FKRP to be located in ER.

Previous attempts to localise endogenous FKRP in muscle have also generated conflicting results. Based on immunohistochemistry on human muscle sections using the same antibody as Matsumoto *et al* (2004) [24], Torelli *et al* (2005) [25] and Dolatshad *et al* (2005) [28] found FKRP to have peri-nuclear localisation. These findings are contrasting our results (this work) as well as the findings of Beedle *et al* (2007) [31] who demonstrated FKRP localisation in the sarcolemma on immunohistochemical sections of mouse muscle cells. Muscle is a heterogeneous tissue and great variation in cellular abundances and distribution patterns of organelles, including the Golgi complex, have been reported; this particularly in relation to fiber-type [32]. Nevertheless, the discrepant results on FKRP intracellular localisation are very intriguing. None of these reports should be disregarded, but rather they should encourage further experiments, using a battery of validated anti FKRP antibodies, to determine whether endogenous FKRP really can exist in the three subcellular muscle cell locations reported until to date; perinucleus [25], [28], sarcolemma [31] and Golgi cisternae (this work).

### FKRP as a homodimer

Several lines of evidence based on non-reducing Western blot analysis, protein cross-linking, pairwise yeast two-hybrid assays and co-immune precipitation (Fig. 2 and 3) demonstrated FKRP-FKRP self-interaction. Interestingly in this respect, whereas FKRP-HA could be co-precipitated with FKRP-Myc from cellular lysates originating from co-transfection experiments, FKRP-HA could not be co-precipitated with FKRP-Myc from a mixture of cellular lysates originating from separate solo transfections with FKRP-HA and FKRP-Myc carrying expression plasmids. This shows that when FKRP-FKRP assembly is once completed in the cellular compartment further FKRP assembly is disallowed; indicating that FKRP-FKRP assembly is a self limiting process that is likely terminated by stoichiometric and steric constrictions such as the complete occupation of interaction interfaces and cysteine residues for possible disulfide bridge formation. Since the FKRP homodimer was clearly sensitive to reduction by DTT, we explored the role of its eight cysteines in dimer formation by substituting them with serine. Replacement of Cys6, but none of the seven other cysteines, disrupted FKRP dimerisation. We conclude, therefore, that FKRP dimers are covalently connected at Cys6, by disulfide linkage.

Important in this respect is that FKRP-FKRP association can take place regardless of the presence of Cys6, but rather through non-covalent interaction as shown in yeast cells. The FKRP constructs used in yeast two-hybrid experiments were devoid of residues 1–31, yet they demonstrated association allowing growth on selective medium. Together with the fact that the severely truncated FKRP-157 construct maintained the property to dimerise, these results strongly indicate that an interaction interface, necessary and sufficient for FKRP homodimer formation, is extending beyond residue 31 and into the N-terminal one third of FKRP polypeptide.

By necessity, since the covalent disulfide bridge is provided by Cys6 alone, the interaction interfaces, as well as the homodimer as a whole, must display two-fold symmetry. Thus, a model can be envisioned in which FKRP homodimerisation is initiated and driven by hydrophobic interactions of which the resulting dimer is subsequently stabilised by a Cys6-Cys6 disulfide bridge.

Recently Lu *et al* (2010) [30] demonstrated N-glycosylation of recombinant FKRP expressed in Chinese Hamster Ovary (CHO) cells. Here we show that both FKRP N-glycosylation sites are occupied and that these glycans are composed of high mannose and/or hybrid oligosaccharides. Furthermore, FKRP N-glycosylation is not required for homodimer or multimer formation. However, it remains unknown whether the N-glycans are required, *in vivo*, for FKRP folding, stability, transport and function.

### FKRP in multimeric complexes

Western blot analysis under protein non-reducing conditions, as well as upon chemical protein cross-linking, showed that FKRP also exists in higher order multimeric complexes. Further dissection of the FKRP-FKRP interaction revealed that whereas the most severely truncated mutant, FKRP-157, devoid of amino acid residues 158–495, displayed no visible multimers, but monomers and dimers only, the two less severely, C-terminally truncated FKRP mutants, FKRP-373 and FKRP-282, maintained the property to express not only monomers and dimers, but clearly also large multimeric complexes. This indicates that whereas the primary FKRP-FKRP dimer interaction interface is located in the N-terminal one-third (1–157), the FKRP involvement in multimer formation must be dependent on amino acid residues belonging to the C-terminal two third - extending from residue 158 and throughout the FKRP polypeptide. Since also large protein complexes (>∼180 kDa) appear to be sensitive to reduction by DTT it is highly likely that other FKRP Cys residues are involved in multimer formation. However, the complex multimer band patterns from the experiments presented here (Fig. 2, 4 and 5) does not allow such Cys residues to be identified at this juncture.

Notably however, at reducing conditions we have repeatedly detected a weak, but distinct, FKRP specific band migrating as ∼170–180 kDa, which disappeared along with the FKRP dimer band of the FKRP-Cys6 mutant (Fig. 2A, 4B and 5C). This suggests that the formation of the ∼170–180 kDa complex depends on a preceding covalent FKRP dimer formation. A strong band of similar molecular weight appeared upon cross-linking with EGS (Fig. 2B). The approximate molecular weight is as expected for a putative FKRP homotrimer (Fig. 4B and 5C). A disulfide-linked FKRP trimer would require the involvement of more than one Cys residue. Therefore, the possible association of an FKRP homodimer with yet a third FKRP polypeptide would have to involve a non-covalent interaction. The composition of multimers, ranging in molecular weight from that of a putative FKRP trimer (∼175 kDa) to several hundred kDas (Fig. 2A and B), likely consist of both homo- and hetero-oligomeric proteins complexes. Their compositions and role in FKRP function remain to be explored.

### FKRP localisation and structure is consistent with Golgi resident type II transmembrane proteins

**Figure 6:**
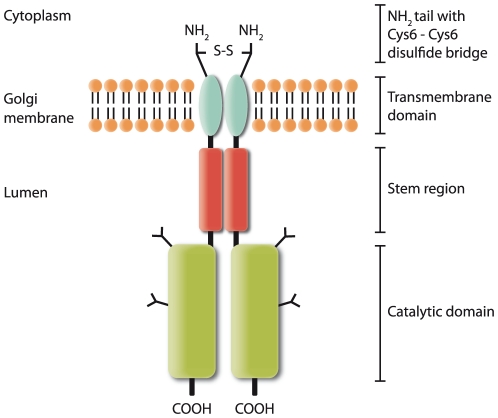
Proposed model of the Golgi resident FKRP dimer; a putative glycosyltransferase. According to the type II transmembrane glycosyltransferase model, the architecture comprises a globular catalytic domain, a stem region, a single pass transmembrane domain and a cytoplasmic N-terminal tail [33], [34], [37]. Based on the present work we suggest that FKRP forms homodimers via an interaction interface that extends through the stem region. The interaction is stabilised by a Cys6-Cys6 disulfide bridge in the N-terminal cytoplasmic tail resulting in a covalently connected FKRP dimer with two-fold symmetry. The catalytic domain is likely to interact with other proteins forming large multimeric structures (not depicted). -S-S-; disulfide bridge, Y; N-glycan of high mannose and/or hybrid type.

The Golgi apparatus is indeed a location that would be expected for an enzyme involved in *de novo* O-glycan synthesis and/or modification. Based on hydrophobicity plots and secondary structure analysis, FKRP was predicted to be a type II transmembrane protein [26]. Most of the knowledge on type II transmembrane proteins has been obtained from studies of N-glycosyltransferases and sulfotransferases. In general Golgi resident type II transmembrane proteins contain a short, cytoplasmically exposed, amino-terminal domain followed by a transmembrane domain, a stem region and a large, globular, catalytic domain facing the luminal side [33], [34]. Furthermore, they are known to form homodimers and heterodimers, as well as homo- and hetero-oligomeric protein complexes with other Golgi resident proteins which function in the same biosynthetic pathway [35], [36], [37], [38]. Accordingly, a model can be envisioned in which the N-terminal transmembrane domains of an FKRP homodimer extend through the Golgi membrane, forming a stabilising Cys6-Cys6 disulfide bond at the cytoplasmic face. A proposed model for the FKRP dimer is depicted in Figure 6.

Interestingly, although its biological significance is yet to be explored, Lu *et al* (2010) [30] demonstrated that recombinant FKRP can be secreted from CHO cells but, in contrast to the putative dimer which was retained in the cell pellet, it was secreted into the supernatant only as a monomer. This monomeric form was presumably devoid of the putative 27 amino acid signal peptide [30]. For such a phenomenon to occur FKRP must be proteolytically cleaved, N-terminally, close to the membrane at the luminal face, releasing FKRP from its N-terminal signal peptide. Thus, their observations do not conflict with our model, but rather they support our finding that Cys6 is the only contributor to a covalent, symmetric, FKRP homodimeric interaction.

In summary, the FKRP localisation and intermolecular interaction determined in this work is consistent with that of a Golgi resident type II transmembrane protein. In the Golgi cisternae FKRP and putative interacting partners will be positioned to modify, or assist the modification of dystroglycan as it passes, *en route*, to the plasma membrane. However, more experiments are required to identify the FKRP substrate (if any) and, moreover, whether FKRP has the capacity to invoke the modification of other proteins besides dystroglycan.

## Materials and Methods

### Human specimens

Ethics Statement: An anonymous human rectus femoris control specimen was provided by the Department of Pathology, University Hospital of North-Norway. The use of this anonymous muscle specimen, for immune electron microscopy, was approved by The Regional Committee for Medical Research Ethics (REK nord). With authorisation in the ACT 2008-06-20 no. 44: the Health Research Act, § 20, REK nord waived the need for consent.

### Antibodies

Anti-FKRP antibodies used for Western blot analyses and/or immune electron microscopy were polyclonal rabbit anti-FKRP antibodies generated against synthetic peptides FKRP107-122: ALDRPAAASRPETYVA, and FKRP425-439: CGVMTKDTWLD HRQDV, according to the double XP program of Eurogentec, Liege, Belgium, and named FKRP207 and FKRP208, respectively. Both antibodies were affinity purified against their corresponding peptide antigens.

Rabbit polyclonal antibodies for the detection of HA (ab13834) and Myc (ab9106) fusion tags were purchased from Abcam, UK. Mouse monoclonal anti-MAP kinase 2 antibody (Erk2 clone 1B3B9) (Upstate/Chemicon, USA), was used as control to assess the stringency in co-immune precipitation (CO-IP) experiments. Secondary antibodies used in Western blot analysis were all conjugated with alkaline phosphatase (AP); these were chicken anti-rabbit antibodies (sc-2967) (Santa Cruz Biotechnology Inc., USA) and rabbit anti-mouse antibodies (Cat No. 81-6722) (Invitrogen, USA).

In immunoelectron microscopy, monoclonal mouse anti-MG160 Golgi complex antibody (AE-6) (ab58826) (Abcam, UK), monoclonal mouse anti-ß-dystroglycan (ß-dys) antibody, (7D11) (sc-33701) (Santa Cruz Biotechnology, USA), and mouse anti- Protein Disulfide Isomerase (PDI) antibody (RL90) (ab2792) (Abcam, UK) served as Golgi [32], sarcolemma [39], [40] and endoplasmic reticulum (ER) markers [41], respectively. The secondary antibodies used were gold conjugated goat F(ab)2 anti-rabbit IgG (5 nm) and goat anti- mouse IgG (10 nm) (British Bio Cell International, UK).

### Immunoelectron microscopy

A human *rectus femoris* muscle biopsy was processed for immunolabelling of sections as described by Tokuyasu (1986) [42]. Briefly, the tissue was fixed in 8% formaldehyde in PBS over night, infiltrated with 2.3 M sucrose, mounted on specimen pins and frozen by immersion in liquid nitrogen. Ultrathin cryosections were made in a Leica EM UC6 Ultra Microtome with a DiATOME diamond knife (DiATOME, Switzerland), thawed on a drop of methylcellulose/sucrose and mounted on carbon coated Formvar films on copper grids. Sections were rehydrated in double-distilled water (dsH_2_O) for 30 min followed by blocking with 1% Cold Water Fish Skin Gelatine (CWFSG) (Sigma G-7765). Immunolabelling was performed according to Griffiths (1993) [43]. For double immunolabelling of FKRP and sub- cellular markers specific to, either Golgi, ER or sarcolemma, sections were incubated with mixtures of primary antibodies at the optimal dilutions determined by single labelling titration experiments. Subsequently after rinsing with PBS, the sections were incubated with a mixture of the appropriate colloidal gold conjugated antibodies. Following rinsing with PBS the specimens were fixed in glutaraldehyde, washed with dsH_2_O and contrasted with methylcellulose and uranyl acetate (9∶1). The specimens were examined with a Jeol 1010 Transmission Electron Microscope (JEOL Ltd., Japan) and micrographs were taken with a Morada Camera system (Olympus Soft Imaging Solutions, Germany).

The following double labelling experiments were performed on human muscle sections: *i)* Rabbit anti-FKRP (FKRP207/208)/mouse anti-MG160, *ii)* rabbit anti-FKRP (FKRP207/208)/mouse anti-ß-dys, *iii)* rabbit anti-FKRP (FKRP207/208)/mouse anti-PDI. Secondary antibodies used in these experiments were goat anti-rabbit, 5 nm gold, in combination with goat anti-mouse, 10 nm gold. To check for possible secondary antibody cross reactivity, control labelling experiments were carried as above, but omitting one of the primary antibodies as follows: *i)* Rabbit anti-FKRP (FKRP207/208). *ii)* Mouse anti- MG160. Secondary antibodies used were the combination of goat anti-rabbit, 5 nm/goat anti-mouse, 10 nm, as described above.

### Pairwise yeast two-hybrid assay

The coding sequence for amino acids 32-494 of human FKRP (hFKRP) (GenBank accession number GI: 209574324; NM_001039885.2) was amplified with primers p5 and p3 (Table S1) and cloned into the bait vector pB27 in-frame with the coding sequence of the LexA DNA binding domain (DBD); pB27 (N-LexA-FKRP32-494-C). pB27 is derived from the original pBTM116 [44]. FKRP amino acids 1–31, thought to contain a trans-membrane domain [26], were omitted as they were suspected to conflict with the yeast two-hybrid assay. By using the *Sfi*I restriction sites on both sides of the bait, the coding sequence for amino acids 32-494 of FKRP32-494 was transferred from the original bait vector pB27 into prey plasmid pP7, in frame with the Gal4 activation domain (AD); pP7 (N-GAL4-FKRP32-494-C). pP7 is derived from the original pGADGH [45]. The correct sequences of the DBD and AD constructs were verified by DNA sequencing with primers as shown in Table S1. FKRP32-494 – FKRP32-494 interaction was tested by co-transformation of the constructs in yeast diploid cells, which were obtained using a mating protocol with L40ΔGal4 (mata) and Y187 (matα) yeast strains [46]. Interaction pairs were tested in duplicate as two independent clones from separate co-transformations. For each interaction, four different dilutions (10^−1^–10^−4^) of the diploid yeast cells, normalised at 5×10^4^ cells, were spotted on selective media. The DO-2 selective medium lacking tryptophan (Trp) and leucine (Leu) was used as a growth control and to verify the co-transformation of bait and prey plasmids. The DO-3 selective medium lacking Trp, Leu and histidine (His) was used to assess FKRP32-494 – FKRP32-494 interaction. Appropriate negative controls included combinations of empty bait and prey plasmids, bait plasmid with FKRP32-494 in combination with empty prey plasmid and *vice versa*. The combination of human Smad3 as bait (GI: 5174512, aa 1–425) and Human Smurf1 as prey (GI: 31317291, aa 150–325, p. N272S, p. F308Y) served as positive control. The yeast two hybrid experiments were carried out in collaboration with Hybrigenics SA services, Paris, France.

### Construction of FKRP expression plasmids

The complete coding sequence of *FKRP*, which is contained in exon 4 of the *FKRP* gene, was PCR amplified from human genomic DNA using primers FKRP 1F and FKRP 1R and subsequently re-amplified with nested primers FKRPOFTO and FKRPOR (Table S1). High fidelity PrimeSTAR Takara HS DNA polymerase (Takara Bio Inc., Japan) was used for all PCR based cloning experiments. The resulting PCR fragment was directionally inserted in conjunction with the CMV promoter of expression plasmid pcDNA3.1D/V5-His-Topo, and subsequently transformed into One Shot TOP10 chemically competent *E. coli* (pcDNA3.1 Directional TOPO Expression Kit) (Invitrogen, USA). Plasmid DNA was purified with either QIAprep Spin Miniprep Kit, Qiagen Plasmid Midi Kit (QIAGEN, Sweden) or NucleoBond Xtra Midi (Marchery-Nagel, Germany), according to manufacturer's specifications. The pcDNA3.1-FKRP expression clone was used as template for further PCR based cloning of FKRP truncation mutants, the generation of FKRP-HA and FKRP-Myc C-terminal fusion proteins and to create FKRP amino acid substitutions. FKRP C-terminal truncation mutants were generated by PCR using primer combinations FKRPOF/FKRP-373, FKRPOF/FKRP-282 and FKRPOF/FKRP-157. FKRP with C-terminal HA and Myc fusions were generated by PCR amplification with primer combinations FKRPOF/FKRP-HA and FKRPOF/FKRP-Myc (Table S1). Resulting PCR fragments were cloned into expression vector pcDNA3.1 by the use of pcDNA3.1/V5-His TOPO TA Expression Kit (Invitrogen, USA) followed by transformation and plasmid DNA purification as explained above. In all the above cloning experiments *FKRP* DNA inserts contained their own translation termination codons (TGA), to prevent translational fusion with the downstream V5-His tags. FKRP Cys→Ser and Asn→Gln amino acid substitutions were generated directly from pcDNA3.1-FKRP plasmid DNA by site directed *in vitro* mutagenesis with primer combinations FKRP-C6S F/FKRP-C6S R, FKRP-C168S F/FKRP-C168S R, FKRP-C191S F/FKRP-C191S R, FKRP-C289S F/FKRP-C289S R, FKRP-C296S F/FKRP-C296S R, FKRP-C317S/C318S F/FKRP-C317S/C318S R, FKRP-C375S F/FKRP-C375S R, FKRP-Q172N F/FKRP-Q172N R, FKRP-Q209N F/FKRP-Q209N R as displayed in Table S1. *In vitro* mutagenesis was carried out using QuickChange Site-Directed Mutagenesis Kit reagents according to manufacturer's specifications (Stratagene, USA). Subsequent to all cloning and *in vitro* mutagenesis, verification of correct insert orientation and *FKRP* sequence was performed by DNA sequencing using flanking and internal sequencing primers as shown in Table S1. Sequencing reactions were carried out with BigDye 3.1 kit reagents (Applied Biosystems, USA). These were separated and detected on an automated sequencer unit (3130xl, Applied Biosystems/Hitachi, USA) and further analysed using the SeqScape v2.5 software (Applied Biosystems, USA).

### Cell culture, transfection and preparation of lysates

African green monkey kidney cells (COS-7) (ATCC: CRL-1651) and Syrian Baby Hamster kidney cells (BHK-21) (ATCC: CCL-10) were used in transfection experiments. COS-7 cells were cultured in Dulbecco's Modified Eagle medium supplemented with GlutaMAX (GIBCO, USA), D-glucose (4.5 mg/ml) and 10% Fetal Bovine Serum (FBS) (GIBCO, USA). BHK-21 cells were cultured in Minimum Essential Medium with Earle's Salts, Glutamax with 5% FBS (GIBCO, USA). Both media were supplemented with 60 U/ml penicillin-G and 60 µg/ml streptomycin (GIBCO, USA). The cells were cultured in 6-well plates and grown to 80–90% confluence prior to transfection. Each well was transfected with 2 µg of plasmid DNA of each expression construct using FuGene HD transfection reagent (Roche, Germany) in Opti-MEM (GIBCO, USA). The cells were harvested after 48 hrs of incubation using Mammalian Protein Extraction Reagent (M-PER, PIERCE, USA) supplemented with Complete Mini EDTA-free protease inhibitor cocktail (Roche, Germany). Whenever stated, the extraction reagent was supplemented with 5 mM N-ethylmaleimide (NEM) (Sigma, Germany). The lysates were cleared by centrifugation at 14000×g for 15 min and protein concentrations were measured by using DC Protein assay kit (BioRad, USA) and a Heigar THERMO_MAX_ Microplate reader.

For protein cross-linking 10 mM Ethylene glycol-bis(SuccinimidylSuccinate) (EGS) (PIERCE, USA) was added immediately to the cleared lysate followed by incubation at room temperature (RT), for 30 min. The reaction was quenched with 35 mM Tris, pH 7.5, for 15 min.

### SDS Page and Western blot analysis

Prior to Sodium Dodecyl Sulfate-Polyacrylamide Gel Electrophoresis (SDS-PAGE) protein samples were added LDS loading buffer as recommended by the supplier (Invitrogen, USA). For reducing gel electrophoresis NuPAGE Sample Reducing agent (10×) (Invitrogen, USA) was added to 1× followed by incubation at 99°C for 5 min or, alternatively, samples were reduced by adding 300–400 mM Dithiothreitol (DTT) (Sigma, Germany), followed by incubation at RT for 30 min. Samples were loaded onto NuPage Novex Bis-Tris 4–12% polyacrylamide gels (Invitrogen, USA). After separation the gel proteins were electrotransferred onto PVDF membranes that were subsequently blocked with 0.1% Tween Phosphate buffered saline (PBST) containing 5%, fat free, dry milk. After incubation with primary and secondary antibodies, membranes were developed by Tropix CDP-*star* (Applied Biosystems, USA). Immunoreactive bands were visualised using a FUJIFILM Luminescent Image Analyzer LAS-3000 (Fuji photo film, Co., LTD, Japan).

### Co-Immune precipitation

Co-immune precipitation was performed using the ProFound Mammalian c-Myc Tag IP/Co-IP Kit (PIERCE, USA). Briefly, clarified cell lysates supplemented with 5 mM NEM and protease inhibitor, containing 600 µg of total protein, were pre-incubated with agarose coupled anti-myc antibody at 4°C for 45 minutes. Subsequent to three washing steps proteins were eluted in 60 µl 2× Non-reducing sample buffer under denaturing conditions (5 min at 99°C). Eluted proteins were subjected to SDS-PAGE at reducing condition followed by Western blot analysis.

### Deglycosylation

Lysates (8–20 µg of protein), prepared from BHK-21 or COS-7 cells transfected with pcDNA3.1-FKRP, were treated with 10× glycoprotein denaturing buffer for 10 min, at 100°C, and then incubated with 1500 units of Endoglycosidase H (Endo H) (NewEngland BioLabs) or 750 units of N-Glycosidase F (PNGase F) (New England BioLabs) for one hour at 37°C.

## Supporting Information

Table S1 **List of primers used for PCR amplification,** ***in vitro*** **mutagenesis, generation of fusion proteins and DNA sequencing.** All primers are shown in 5′to 3′direction. Sequences that encode HA or Myc tags are shown in bold. Stop codons introduced in reverse primers (R) are underlined. Nucleotides introduced for site directed mutagenesis are underlined. *SpeI* and *PacI* restriction sites are shown in lower case.
(DOCX)
