# Supplementary material for: Fukutin-Related Protein Resides in the Golgi Cisternae of Skeletal Muscle Fibres and Forms Disulfide-Linked Homodimers via an N-Terminal Interaction
Source: PLoS One. 2011 Aug 23;6(8):e22968. doi: 10.1371/journal.pone.0022968 (PMC3160285; doi:10.1371/journal.pone.0022968)
Supplement: Table S1 — List of primers used for PCR amplification, in vitro mutagenesis, generation of fusion proteins and DNA sequencing. All primers are shown in 5′to 3′direction. Sequences that encode HA or Myc tags are shown in bold. Stop codons introduced in reverse primers (R) are underlined. Nucleotides introduced for site directed mutagenesis are underlined. SpeI and PacI restriction sites are shown in lower case. (DOCX) [file pone.0022968.s001.docx]

**Table S1:**

Primers used for the cloning of human FKRP:

FKRP F1 - GACAATCAGCTGCTGCCTTCCC

FKRP R1- ACAGAGCTTCTCCACATCCAG

FKRPOF - CCATGCGGCTCACCCGCTGCCAG

FKRPOFTO - CACCATGCGGCTCACCCGCTGCCAGG

FKRPOR - CTTCAGCCGCTTCCCGTCAGACTCA

Primers used to generate FKRP C-terminal truncations:

FKRP-157R: TCAGGCCACCAGACGTGCGCTTCCT

FKRP-282R: TCACCCGCCTTCCCAGCTCACTAGG

FKRP-373R: TCAGCCCACGTCCTCCAAGTAGATG

Primers used to create C-terminal FKRP-Myc and FKRP-HA fusion proteins:

**FKRP-Myc**: TCA **CAG GTC CTC CTC GCT GAT CAG CTT CTG CTC** GCC GCT TCC CGT CAG ACT CA

**FKRP-HA:** TCA **GGC GTA GTC GGG CAC GTC GTA GGG GTA** GCC GCT TCC CGT CAG ACT CA

Primers used to create FKRP amino acid substitutions:

FKRP-C6 F: CGG CTC ACC CGC T**C**C CAG GCT GCC CTG

FKRP-C6 R: CAG GGC AGC CTG G**G**A GCG GGT GAG CCG

FKRP-C168S F: C AAC CCT GCC AGG T**C**C CTG GCC CTG AAC

FKRP-C168S R: GTT CAG GGC CAG G**G**A CCT GGC AGG GTT G

FKRP-C191S F: GCC GCG CCC CGC T**C**C GAC GCC CTG GAC

FKRP-C191S R: GTC CAG GGC GTC G**G**A GCG GGG CGC GGC

FKRP-C289S F: GAG TGG TTC GGC T**C**C AAC AAG GAG ACC

FKRP-C289S R: GGT CTC CTT GTT G**G**A GCC GAA CCA CTC

FKRP-C296S F: GAG ACC ACG CGC T**C**C TTC GGA ACC GTG

FKRP-C296S R: CAC GGT TCC GAA G**G**A GCG CGT GGT CTC

FKRP-C317S/C318S F: G ACG CCC CCC T**C**C T**C**C CTG CGC GCG CTG

FKRP-C317S/C318S R: CAG CGC GCG CAG G**G**A G**G**A GGG GGG CGT C

FKRP-C375S F: GAC GTG GGC AAC T**C**C GAG CAG CTG CGG

FKRP-C375S R: CCG CAG CTG CTC G**G**A GTT GCC CAC GTC

FKRP-Q172N F: GC CTG GCC CTG **C**A**G** GTC AGC CTG CG

FKRP-Q172N R: CG CAG GCT GAC **C**T**G** CAG GGC CAG GC

FKRP-Q209N F: CGC GAC CTC TTC **C**A**G** CTC TCG GCG CCC

FKRP-Q209N R: GGG CGC CGA GAG **C**T**G** GAA GAG GTC GCG

Primers used for the cloning of FKRP32-494 into the bait vector pP27 (N-LexA-bait-C):

5p : (*SpeI*)cggactagtTAGGAATTCCCGGGCCCGGGGG

3p : (*PacI*)gccttaattaaTCAGCTTCCCGTCAGACTCAGCAG

Additional FKRP sequencing primers:

For FKRP expression constructs:

T7 F: TAATACGACTCACTATAGGG

BGH R: TAGAAGCGACAGTCGAGG

H-FKRP 388bp: GGTACTAGGGCCACAAACTC

FKRP-2F: GGCACCAGCCTCTTTCTGCAG

FKRP-2R: GTCTCCTTGTTGCAGCCGAACCA

For pP27:FKRP:

5p: AGCTTCACCATTGAAGGGCT

3p: CATAAGAAATTCGCCCGG

For p7:FKRP:

5p: CGCGTTTGGAATCACTACAGGG

3p: TGCACAGTTGAAGTGAACTTGCGGGGTT

Internal FKRP sequencing primers:

GAACGTCAGCCTGCGAGAGT

CAGCTGCTGGACTTGACCTT

CTCTACGAGGAGCGCTGGAC
